# Comparing radiomic classifiers and classifier ensembles for detection of peripheral zone prostate tumors on T2-weighted MRI: a multi-site study

**DOI:** 10.1186/s12880-019-0308-6

**Published:** 2019-02-28

**Authors:** Satish E. Viswanath, Prathyush V. Chirra, Michael C. Yim, Neil M. Rofsky, Andrei S. Purysko, Mark A. Rosen, B Nicolas Bloch, Anant Madabhushi

**Affiliations:** 1 Department of Biomedical Engineering, Case Western Reserve University, Cleveland, OH USA; 2 College of Medicine, Northeast Ohio Medical University, Rootstown, OH USA; 3 Department of Radiology, UT Southwestern Medical Center, Dallas, TX USA; 4 Department of Radiology, Cleveland Clinic, Cleveland, OH USA; 5 Department of Radiology, Hospital of the University of Pennsylvania, Philadelphia, PA USA; 6 Department of Radiology, Boston University School of Medicine, Boston, MA USA

**Keywords:** Classifiers, Radiomics, Prostate cancer, MRI, Comparison

## Abstract

**Background:**

For most computer-aided diagnosis (CAD) problems involving prostate cancer detection via medical imaging data, the choice of classifier has been largely ad hoc, or been motivated by classifier comparison studies that have involved large synthetic datasets. More significantly, it is currently unknown how classifier choices and trends generalize across multiple institutions, due to heterogeneous acquisition and intensity characteristics (especially when considering MR imaging data). In this work, we empirically evaluate and compare a number of different classifiers and classifier ensembles in a multi-site setting, for voxel-wise detection of prostate cancer (PCa) using radiomic texture features derived from high-resolution in vivo T2-weighted (T2w) MRI.

**Methods:**

Twelve different supervised classifier schemes: Quadratic Discriminant Analysis (QDA), Support Vector Machines (SVMs), naïve Bayes, Decision Trees (DTs), and their ensemble variants (bagging, boosting), were compared in terms of classification accuracy as well as execution time. Our study utilized 85 prostate cancer T2w MRI datasets acquired from across 3 different institutions (1 for discovery, 2 for independent validation), from patients who later underwent radical prostatectomy. Surrogate ground truth for disease extent on MRI was established by expert annotation of pre-operative MRI through spatial correlation with corresponding *ex vivo* whole-mount histology sections. Classifier accuracy in detecting PCa extent on MRI on a per-voxel basis was evaluated via area under the ROC curve.

**Results:**

The boosted DT classifier yielded the highest cross-validated AUC (= 0.744) for detecting PCa in the discovery cohort. However, in independent validation, the boosted QDA classifier was identified as the most accurate and robust for voxel-wise detection of PCa extent (AUCs of 0.735, 0.683, 0.768 across the 3 sites). The next most accurate and robust classifier was the single QDA classifier, which also enjoyed the advantage of significantly lower computation times compared to any of the other methods.

**Conclusions:**

Our results therefore suggest that simpler classifiers (such as QDA and its ensemble variants) may be more robust, accurate, and efficient for prostate cancer CAD problems, especially in the context of multi-site validation.

## Background

Pattern recognition approaches for distinguishing between object classes (diseased versus normal or cancerous versus benign) on bioinformatics [1, 2] or medical imaging [3, 4] data typically involve first extracting informative features, which are then used to train a machine learning classifier. In the case of medical imaging data, depending on the specific classes to be discriminated, a variety of computerized image-derived (i.e. *radiomic*) features have been proposed and evaluated [5–7]. A problem that has perhaps not received as much attention is the choice of classifier scheme for a particular computer aided detection (CAD) problem. The advent of ensemble schemes (bagging [8], boosting [9]) to overcome known shortcomings of classifier algorithms with respect to bias and variance [10] have further expanded the choices available when choosing an optimal classifier.

While several classifier comparison studies [11–16] have been reported using large standardized datasets, there has been some lack of concordance with regard to their recommendations for choice of optimal classifier scheme or how classifier trends generalize as in the presence of noise. Most recently, one of the largest comparison studies evaluated 179 classifiers on the popular UCI machine learning repository [17] and determined that random forests may be the most effective choice for most problems [18]. Thus far, medical imaging CAD studies [3, 4, 19] have also arrived at similar conclusions when identifying the optimal classifier scheme or when reporting trends between classifiers for a specific problem. In comprehensive comparisons of 12 different classifier methods on large databases of lung cancers as well as head & neck cancers when using radiomic features (including independent training and validation cohorts), it was reported that the random forest classifier yielded the best predictive performance in both problems [20, 21]. Notably, both these studies also acknowledged that the choice of classifier method had the most dominant effect on predictive performance (i.e. it was a larger source of performance variability as compared to feature selection method or size of cohort).

**Fig. 1:**
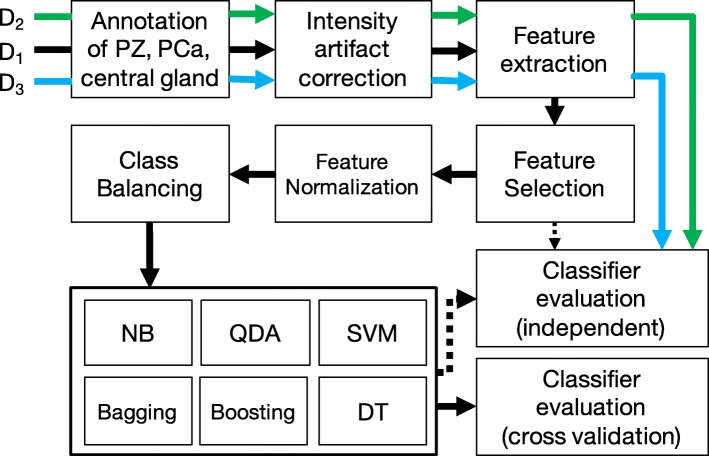
Overview of experimental workflow. Arrow colors show how training (*D*_1_, black) and independent validation cohorts (*D*_2_ and *D*_3_ in green and blue) go through different stages

In this work we aim to compare classifier performance in the specific context of voxel-wise detection of tumors in the peripheral zone (PZ) of the prostate via “radiomic” texture-based features derived from T2w MRI. While a variety of approaches have been proposed for prostate cancer CAD [22] recently, they have typically been evaluated via cross-validation using data from a single site or scanner. While acquisition-related noise artifacts and acquisition protocols are relatively homogeneous and well understood in the single-center setting, these issues are less studied when considering imaging data from multiple institutions. Multi-site data suffers from differences in scanners, acquisition protocols, and resolution differences - all of which can affect classifier performance, and hence choice of classifier, significantly. To address this question, we will perform an empirical examination of how classifier trends and performance generalize for prostate cancer (PCa) detection on MRI data that has been acquired from across multiple different institutions and scanners. The classifiers we will consider include discriminant analysis (DA) [23], support vector machines (SVMs) [24], naïve Bayes [25], and decision trees (DTs) [26], as well as their bagging and boosting variants; thus covering most popular families of classifier methods. We will attempt to identify the optimal classifier for prostate cancer CAD in terms of cross-site detection accuracy, while considering computational complexity as well. The overall workflow and experimental design of our paper is depicted in Fig. 1.

## Methods

### Data description

A total of 85 patient datasets were considered in this study. These were retrospectively obtained after de-identification from 3 different institutions, under previous IRB-approved research protocols. For the current study, informed consent was waived by the IRB because the data was obtained retrospectively and did not include any protected health information, by the University Hospitals IRB. All patients included had first been confirmed to have prostate cancer via positive core needle biopsies and later underwent a radical prostatectomy. Prior to surgery, all patients had been imaged via MRI using a combined torso-phased array and endorectal coil, at their respective institutions. Further details of the imaging acquisition at each institution are summarized in Table 1. Note that *D*_1_ was utilized for discovery and optimization of the classifiers alone. *D*_2_ and *D*_3_ were then used for independent evaluation and validation of classifier performance and trends.

**Table 1:** Summary of multi-site prostate T2w MRI data used in this study

Site	[x,y,z] voxel dimensions (mm)	Magnetic field strength (T)	TR/TE (ms)	Number of datasets
*D*_1_	[0.27,0.27,2.20]	3	4216 - 8266/ 155 - 165	16
*D*_2_	[0.41,0.41,3.00]	3	2840 - 7500/ 107 - 135	13
*D*_3_	[0.27,0.27,2.96]	3	4754/115	56

*D*_1_ was used as the discovery cohort, while *D*_2_ and *D*_3_ were used as independent validation cohorts

### Expert annotation of central gland, PZ, and PCa extent on T2w MRI

For all 85 datasets considered, the central gland and the PZ were annotated on the the axial endorectal T2w MRI image by a radiologist (a different expert annotated data from each institution). Regions of cancer extent within the PZ were annotated as follows:

*Cohorts* *D*_1_, *D*_2_: As the radical prostatectomy specimens had been processed as whole-mount histology sections at these sites, it was possible to identify corresponding WMHS and MRI sections. Each pair of corresponding WMHS and MR images were first affinely aligned to enable correction of large translations, rotations, and differences in image scale. A non-linear alignment of WMHS and MR images was then performed via fully automated, non-linear hierarchical (multi-scale) B-spline registration driven by a higher-order variant of mutual information [27]. This allowed for spatial mapping of pathologic annotations of PCa extent onto T2w MRI, which were further examined and manually corrected for registration artifacts (as required) by the radiologist for that site.

*Cohort* *D*_3_: As only low-resolution photographs of digitized whole-mount sections were available from this site, these had to be visually correlated with corresponding MRIs by a radiologist. Based on this information, they annotated PCa extent on the T2w MR images.

**Fig. 2:**
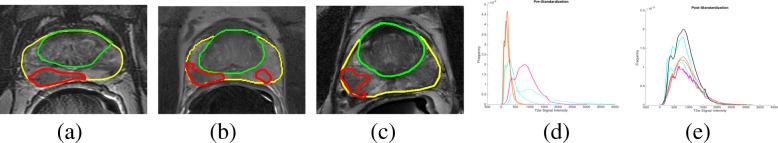
Annotated prostate T2w MRI midgland sections from (**a**) *D*_1_, (**b**) *D*_2_, (**c**) *D*_3_; overlaid with delineations of peripheral zone (yellow), PCa (red), and central gland (green). Also shown are representative T2w intensity distributions from different sites (**d**) before standardization, and (**e**) after correcting intensity drift, where colors correspond to: *D*_1_ (orange), *D*_2_ (cyan), *D*_3_ (pink), template (black)

For the purposes of this study, only PZ regions within the midgland region of the prostate were considered in all 85 datasets. This was to ensure that there was maximum confidence in the PCa annotations as well as consistency in non-tumor and tumor characteristics across sites. Any regions within the PZ that were not annotated as cancer were thus considered to be “non-tumor” regions. In total, we had 116 prostate tumors annotated across 85 patient datasets. Representative 2D T2w MRI midgland sections from each site together with annotations for central gland, PZ, and PCa are depicted in Fig. 2a–c.

### Post-processing of T2w MRIs to account for intensity-based artifacts

The prostate ROI was corrected for known acquisition-based intensity artifacts; bias field inhomogeneity [28] and intensity non-standardness [29]. The effects of bias field occur due to the usage of an endorectal probe [28], and manifest as a smooth variation of signal intensity across the T2w MR image. Bias field has been shown to significantly affect the automated classification of tissue regions [30], and was corrected for via the popular N3 algorithm [31]. Intensity non-standardness [29] refers to the issue of MR signal “intensity drift” across different imaging acquisitions, resulting in MR signal intensities lacking tissue-specific numeric meaning between images obtained on different scanners, despite using the same MRI protocol for the same body region. This can be seen the widely varying intensity distributions depicted in Fig. 2d (different colors correspond to different sites). This was corrected using an automated implementation of the method presented by Nyul et al. [29], whereby the signal intensity histograms across different patient MRI studies were non-linearly aligned to a common template. As a result of standardization, the distributions in Fig. 2e appear to be more consistent and aligned to the common template (shown in black).

Each of the 85 datasets were post-processed for these artifacts independent of each other. We denote $\mathcal {C} = (C,f)$ as representing the corrected, standardized prostate ROI comprising voxels (samples) *c*∈*C*, where *f*(*c*) represents the MR image intensity value at voxel *c*. The set of voxels in PCa regions (as annotated by experts) are denoted as *G*(*C*)={*c*|*l*(*c*)=1} (also called the *target class*, denoted *ω*_+1_). Table 2 summarizes commonly used notation and symbols appearing in this paper.

**Table 2:** Summary of commonly used notation and symbols in this paper

*c*	Samples in set *C*
*n*	Number of samples in *C*
**F**(*c*)	N-dimensional (texture) feature vector
$\mathcal {F}$	Set of all feature vectors
*l*(*c*)	Class label of sample *c*
*ω*_+1_,*ω*_−1_	Classes associated with *l*(*c*)=1,*l*(*c*)=0
**h** ^*β*^	Classifier, *β*∈{*QDA,SVM,Bay,DT*}
$h^{\beta }_{t}$	Component classifier within **h**^*Bag*,*β*^,**h**^*Boost*,*β*^
**h** ^*Bag*, *β*^	Bagged classifier
**h** ^*Boost*, *β*^	Boosted classifier

### Extracting PZ tumor specific radiomic texture features from T2w MRI

It has previously been demonstrated that PZ tumor appearance on T2w MRI may be specifically modeled by image texture (radiomic) features [32, 33]; many of which have been widely used in the prostate cancer CAD literature [22]. A total of 116 image features corresponding to 4 different types of texture were extracted, including Gabor [34] and Haar [35] wavelet features, as well as first and second order texture [36, 37] features. After feature extraction, every voxel *c*∈*C* is associated with a 116-dimensional feature vector denoted $\widehat {\mathbf {F}}(c) = \{f_{1}(c), f_{2}(c),\dots, f_{116}(c)\}$, for every *c*∈*C*.

In order to determine radiomic features specific to PZ PCa regions, feature selection was performed using the minimum Redundancy Maximum Relevance (mRMR) method [38], using voxel-wise features and corresponding voxel-wise labels for tumor and non-tumor regions from site *D*_1_ alone. The mRMR scheme attempts to simultaneously optimize two distinct criteria: (a) selecting features that have the maximal mutual information (MI) with respect to the corresponding set of labels, and (b) that selected features are those have the minimum MI with respect to each other. Use of mRMR ensured that bias towards a particular classifier was prevented as the feature selection step utilized an independent objective function (MI). This is in direct contrast to forward or backward feature selection [39] where the classifier is more integral to the selection process. The result of mRMR feature selection was a subset of 25 voxel-wise radiomic features characterizing PZ PCa appearance (denoted **F**(*c*), complete listing in Appendix A).

### Classifier construction

The feature set **F**(*c*) was input to the different classification algorithms and their ensemble variants (summarized in Table 3). The specific steps for classifier construction are described below. All classifiers were constructed using datasets from discovery site *D*_1_ alone.

**Table 3:** Machine learning classification algorithms evaluated in this work, together with their associated parameters and notation

QDA [23]	**h** ^*QDA*^	-	MATLAB
	**h**^*Bag, QDA*^, **h**^*Boost, QDA*^	*T*=50	MATLAB
SVM [24, 49]	**h** ^*SVM*^	*Ω*,*λ*	LIBSVM
	**h**^*Bag, SVM*^, **h**^*Boost, SVM*^	*Ω*,*λ*,*T*=50	LIBSVM [49], MATLAB
Naïve Bayes [50]	**h** ^*Bay*^	-	MATLAB
	**h**^*Bag, Bay*^, **h**^*Boost, Bay*^	*T*=50	MATLAB
Decision Trees [26]	**h** ^*DT*^	-	C4.5
	**h**^*Bag, DT*^, **h**^*Boost, DT*^	*T*=50	MATLAB TreeBagger, PBTs [51]

SVM parameters include *Ω* (trade-off between training error and model complexity) and *λ* (normalization factor for inputs), which are determined via a grid search strategy. For ensemble approaches, *T* refers to the number of component classifiers

#### Feature normalization

Normalization of radiomic features ensures that different feature values lie in a comparable range of values when input to a classifier. Given a feature vector **F**(*c*), this can be done for each *f*_*i*_(*c*)∈**F**(*c*) as follows,

1 $$ f_{i}(c) = \frac{f_{i}(c)-\mu_{\mathbf{i}}}{\sigma_{i}},   $$

where *μ*_*i*_ is the mean and *σ*_*i*_ is the mean absolute deviation (MAD) corresponding to feature *i,i*∈{1,…,*N*}. As a result of normalization, ∀*c*∈*C*, each feature in **F**(*c*) was transformed to have a mean of 0 and a MAD of 1. Note that radiomic features from *D*_2_ and *D*_3_ were normalized with respect to the mean and MAD of corresponding radiomic features from *D*_1_.

#### Class balancing

A significant issue when training a supervised classifier is the *minority class problem* [40], wherein the target class (in this study *ω*_+1_) has significantly fewer samples compared to the other class (*ω*_−1_), i.e. |*ω*_+1_|≪|*ω*_−1_|. Weiss et al. [40] and Doyle et al. [41] previously showed that using an imbalanced training set will likely result in a lower classifier accuracy compared to balanced training sets (|*ω*_+1_|=|*ω*_−1_|). The class balance problem was addressed for each of the base classifiers, as well as their ensemble variants. Note that class balancing and data sub-sampling was only applied to the training data in each case.

- *QDA, DTs*: For classifiers corresponding to these two families (**h**^*QDA*^, **h**^*Bag,QDA*^, **h**^*Boost,QDA*^, **h**^*DT*^, **h**^*Bag,DT*^, **h**^*Boost,DT*^), class imbalance was accounted for by randomized under-sampling of the majority class (*ω*_−1_) such that |*ω*_+1_|=|*ω*_−1_|, i.e. an equal class balance was maintained when training the classifier.
- *SVMs*: Due to the complex nature of this algorithm, not only did a class balance have to be ensured in the training data, but the number of samples (voxels) used to train the classifier had to be reduced to ensure convergence within a reasonable amount of time. When training an SVM classifier, an equal number of voxels (not less than 0.7×|*ω*_+1_|) were randomly sub-sampled from both *ω*_+1_ and *ω*_−1_ classes to form the training dataset. The number of samples was empirically decided based on a trade-off between execution time, classifier accuracy, and memory constraints specific to the SVM classifier. This procedure was adopted for all classifiers in the SVM family (**h**^*SVM*^,**h**^*Bag,SVM*^,**h**^*Boost,SVM*^).
- *Naïve Bayes*: Training of the naïve Bayes classifier was implemented by directly estimating distributions for each of the classes, *ω*_+1_ and *ω*_−1_, based on all the samples present. Such an estimate is most accurate when the maximal number of samples is utilized in calculating the distribution. Thus, no sub-sampling of the data was performed when constructing these classifiers (**h**^*Bay*^,**h**^*Bag,Bay*^,**h**^*Boost,Bay*^).

#### Classifier training

All classifiers were trained and evaluated via 2 approaches:

- *Three Fold Cross Validation (3FCV)*: In a single cross-validation run for 3FCV, all the datasets from cohort *D*_1_ were divided into 3 random subsets (comprising 6, 5, and 5 studies). 2 subsets were considered for training while the third was held out for independent testing. This process was repeated until all 3 subsets were classified at least once within each cycle. Each cycle was repeated 25 times. Within each cross-validation cycle, the classification results were cumulatively evaluated over all testing results to obtain a single AUC value, in addition to estimating lower and upper bounds on the AUC.
- *Multi-Site Validation (MSV)*: The entire discovery cohort *D*_1_ was utilized to train a classifier model. This trained model was then evaluated for detecting PCa on a voxel-wise basis within the PZ for each dataset from validation cohorts *D*_2_ and *D*_3_. Classification results were evaluated to obtain a per-dataset AUC value, which were then averaged to obtain a per-cohort AUC value (and standard deviation).

Note that feature selection and classifier construction were done separately for each set of training data so constructed, with corresponding testing data only used for evaluation of classifier performance. All classifications were performed and evaluated on a per-voxel basis.

### Evaluation of voxel-wise PCa classifiers

#### Classifier accuracy

In the case of **h**^*QDA*^(*c*), **h**^*SVM*^(*c*), **h**^*Bay*^(*c*), **h**^*Bag*,*β*^(*c*), **h**^*Boost*,*β*^(*c*), *β*∈{*QDA,Bay,SVM,DT*}, which yield a probabilistic result (SVM hard decisions were also converted to a probabilistic result [42]), a binary prediction result at every *c*∈*C* can be obtained by thresholding the associated probability value **h**(*c*)∈ [ 0,1]. These classifier can be evaluated via Receiver Operating Characteristic (ROC) curves [25], representing the trade-off between classification sensitivity and specificity of voxel-wise PCa detection. In the case of **h**^*DT*^(*c*), the output is a single hard partitioning of the sample *c*∈*C* into one of the two classes under consideration. In this case, a single detection result is calculated at a single threshold, based on which a single value for specificity and sensitivity can be calculated. It is assumed that the remaining points on the ROC curve for **h**^*DT*^(*c*) are at [0,0] and [1,1], hence allowing the construction of a pseudo-ROC curve.

ROC curves were visualized for the training cohort *D*_1_ by fitting a smooth polynomial through each set of sensitivity and specificity values calculated for each of the 3FCV runs, and averaging over all the curves generated for each classifier considered. The area under the ROC curve (AUC) was used as a measure of classification performance for both 3FCV and MSV, as is commonly reported in the literature [20, 22, 43].

While analyzing ROC results, the 12 classifiers were segregated into 3 groups, (1) single classification strategies (comprising **h**^*QDA*^, **h**^*Bay*^, **h**^*SVM*^, **h**^*DT*^), (2) bagging strategies (comprising **h**^*Bag, QDA*^, **h**^*Bag, Bay*^, **h**^*Bag, SVM*^, **h**^*Bag, DT*^), and (3) boosting strategies (comprising **h**^*Boost, QDA*^, **h**^*Boost, Bay*^, **h**^*Boost, SVM*^, **h**^*Boost, DT*^). Classifier comparisons were first made within each group (e.g. which of the single classification strategies **h**^*QDA*^, **h**^*Bay*^, **h**^*SVM*^, and **h**^*DT*^ performed best), following which classifier performance across groups was examined. These trends were first examined for the 3FCV results, followed by examining them separately for MSV results. When evaluating MSV results, comparisons were also made to determine how well classifier performance and trends generalized across the 3 sites.

#### Statistical testing

For the 3FCV procedure, each classifier yielded a set of 25 AUC values (corresponding to each cycle of the procedure). For the MSV procedure, each classifier yielded 13 and 56 AUC values (corresponding to the number of datasets in cohorts *D*_2_ and *D*_3_).

Multiple comparison testing to determine statistically significant differences in performance within groups (e.g. between all of **h**^*QDA*^, **h**^*Bay*^, **h**^*SVM*^, **h**^*DT*^) was performed using the Kruskal—Wallis (K-W) one-way analysis of variance (ANOVA) [44]. The K-W ANOVA is a non-parametric alternative to the standard ANOVA test which does not assume normality of the distributions when testing. The null hypothesis for the K-W ANOVA was that the populations from which the AUC values originate have the same median. Based off the results of a K-W ANOVA, multiple comparison testing was performed to determine which groups (single classification strategies, bagging strategies, boosting strategies) show significant differences in performance. Similar multiple comparison testing was also performed to determine significant differences in classifier performance between sites.

Pairwise comparisons were performed for classifiers across groups (e.g. between **h**^*QDA*^ and **h**^*Bag, QDA*^) to identify statistically significant differences in performance. This was done using the non-parametric Wilcoxon rank-sum test [44]. The null hypothesis in such a case was that there were no statistically significant differences in AUC values between the 2 classifiers being compared.

The Bonferroni correction [44] was applied to correct the *p*-value within all statistical comparisons considered (whether pairwise or other).

#### Computation time

For each of the classifiers compared, **h**^*β*^,**h**^*Bag*,*β*^, **h**^*Boost*,*β*^, *β*∈{*QDA,Bay, SVM,DT*}, the total amount of time required during each 3FCV cycle of *D*_1_ for (i) classifier construction, and (ii) for executing the constructed classifier on testing data, was recorded in seconds. The execution time for each classifier was averaged over all cross-validation runs. All algorithms were implemented and evaluated using built-in or publicly available implementations for MATLAB *Ⓡ*9.10 (The Mathworks, MA).

## Results

### Classification accuracy

**Fig. 3:**
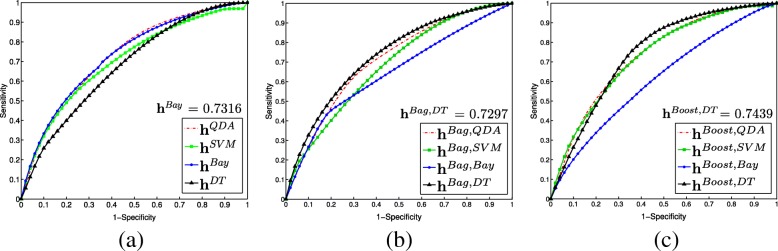
(**a**)-(**c**) ROC curves obtained by averaging over 25 runs of 3FCV for PCa classification in the PZ for discovery cohort *D*_1_. In each graph different colors correspond to different classifier strategies: (**a**) **h**^*QDA*^ (red), **h**^*SVM*^ (green), **h**^*Bay*^ (blue), **h**^*DT*^ (black); (**b**) **h**^*Bag, QDA*^ (red), **h**^*Bag, SVM*^ (green), **h**^*Bag, Bay*^ (blue), **h**^*Bag, DT*^ (black), and (**c**) **h**^*Boost, QDA*^ (red), **h**^*Boost, SVM*^ (green), **h**^*Boost, Bay*^ (blue), **h**^*Boost, DT*^ (black)

**Fig. 4:**
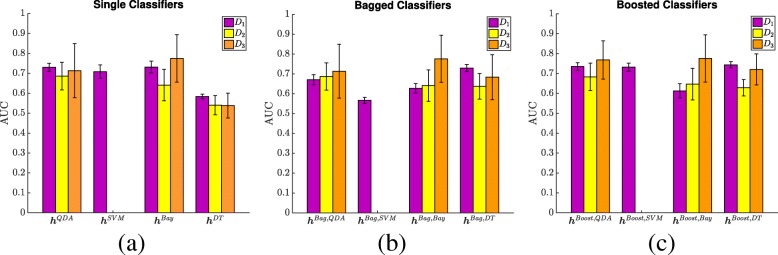
Bar plots comparing voxel-wise PCa classifier performance between *D*_1_ (purple), *D*_2_ (yellow), and *D*_3_ (orange) for (**a**) single classifiers, (**b**) bagged classifiers, and (**c**) boosted classifiers. As all variants of the SVM classifier only converged in the training cohort *D*_1_; corresponding bars for *D*_2_ and *D*_3_ have been omitted. Error bars correspond to the standard deviation in AUC across each cohort

Figure 3 shows average ROC curves (over all 3FCV runs) for voxel-wise PCa classification performance in the PZ, using the training cohort *D*_1_. Figure 4 depicts bar plots of AUC values (with standard deviations as error bars) comparing voxel-wise PCa classification performance in the training cohort *D*_1_ (purple bars) against the 2 independent validation cohorts *D*_2_ (yellow bars) and *D*_3_ (orange bars). Note that both single and ensemble SVM classifiers only reached convergence in *D*_1_ but not in *D*_2_ and *D*_3_, and thus had to be omitted. This was likely due to the large number of training samples (voxels), which in conjunction with the grid search optimization of SVMs, caused these methods to error out before completion.

#### Comparing single classifier strategies

Figure 3a shows that all three of **h**^*QDA*^ (red), **h**^*Bay*^ (blue), and **h**^*SVM*^ (green) performed comparably for PCa classification in the PZ in *D*_1_, with no statistically significant differences in their AUC values. **h**^*DT*^ (black) demonstrated significantly poorer performance than **h**^*QDA*^, **h**^*Bay*^, and **h**^*SVM*^ in a multiple comparison test of 3FCV AUC values (based off K-W ANOVA) in *D*_1_.

As shown in Fig. 4a, this trend continued to hold in cohorts *D*_2_ and *D*_3_, where **h**^*DT*^ again performed significantly worse than **h**^*QDA*^ and **h**^*Bay*^. In fact, **h**^*DT*^ performed significantly worse in *D*_2_ and *D*_3_ compared to *D*_1_, with an ≈8*%* drop in AUC in independent validation. As DTs often performed on par with guessing (AUC range 0.54-0.58), they may be extremely suboptimal as a single classifier, and may be best used within ensembles (as typically formulated [10]). When comparing classifier performance between sites, all the classifiers performed worse in *D*_2_ than in *D*_3_ (though this was not always statistically significant).

#### Comparing bagged classifier strategies

Figure 3b demonstrates that **h**^*Bag,DT*^ yielded a significantly improved PCa classification performance compared to all of **h**^*Bag,QDA*^, **h**^*Bag,SVM*^, and **h**^*Bag*,*Bay*^, in *D*_1_. Additionally **h**^*Bag,SVM*^ was the worst performing bagged classifier, with significantly lower AUC values compared to all of **h**^*Bag,QDA*^, **h**^*Bag,Bay*^, and **h**^*Bag,DT*^.

Figure 4b depicts the change in these trends for independent evaluation of the bagged classifiers. In *D*_2_, **h**^*Bag,QDA*^, **h**^*Bag,Bay*^, and **h**^*Bag,DT*^ demonstrated no significant differences in performance. By contrast, **h**^*Bag,Bay*^ performed significantly better in *D*_3_ compared to either of **h**^*Bag,QDA*^ and **h**^*Bag,DT*^. Notably, the performance of **h**^*Bag,DT*^ was significantly better in the training cohort *D*_1_ than in the validation cohorts *D*_2_ and *D*_3_ (corresponding to 6−10*%* higher AUC in 3FCV evaluation). Conversely, while performance of **h**^*Bag,QDA*^ and **h**^*Bag,Bay*^ on *D*_1_ were reflective of their performance on *D*_2_ (i.e. no significant differences), both these classifiers showed a significant improvement in *D*_3_.

A significant improvement in performance for **h**^*Bag,SVM*^ and **h**^*Bag,DT*^ was seen on *D*_1_, compared to using **h**^*SVM*^ or **h**^*DT*^ (Wilcoxon test *p*<0.01). This observation held when examining corresponding results on *D*_2_ and *D*_3_, where **h**^*Bag,DT*^ performed significantly better than **h**^*DT*^ (Wilcoxon test *p*<<0.01). This result can be explained by the fact that SVMs and DTs are known to have high variance [45], which would imply they are most likely perform better in conjunction with bagging.

#### Comparing boosted classifier strategies

All 3 of **h**^*Boost,SVM*^, **h**^*Boost,DT*^, and **h**^*Boost,QDA*^ showed no significant differences in performance in 3FCV evaluation in *D*_1_ (Fig. 3c), though **h**^*Boost,Bay*^ performed significantly worse by comparison. As seen in Fig. 4c, these trends did not hold in *D*_2_ and *D*_3_, where **h**^*Boost,Bay*^ performed on par with the other boosted classifiers. When comparing classifier performance between sites, **h**^*Boost,QDA*^ and **h**^*Boost,DT*^ performed worse in *D*_2_ than in *D*_3_ (though this was not always statistically significant).

**h**^*Boost,QDA*^, **h**^*Boost,DT*^, and **h**^*Boost,SVM*^ yielded a marginal but significantly improved performance compared to **h**^*Bag,QDA*^, **h**^*Bag,DT*^, and **h**^*Bag,SVM*^ on *D*_1_. However this trend did not hold in *D*_2_ and *D*_3_, where the bagged and boosted variants of the different classifiers did not perform significantly differently from each other.

**h**^*Boost,DT*^ did yield a significantly improved performance compared to **h**^*DT*^ in all 3 cohorts (as did **h**^*Bag,DT*^). While optimal performance of bagging is highly dependent on the component classifiers exhibiting high variance [8], boosting is dependent on the component classifiers having low bias [45]. Thus classifiers which enjoy both these advantages (such as DTs) thus show significant performance improvements when combined within an ensemble.

### Classifier execution time

**Fig. 5:**
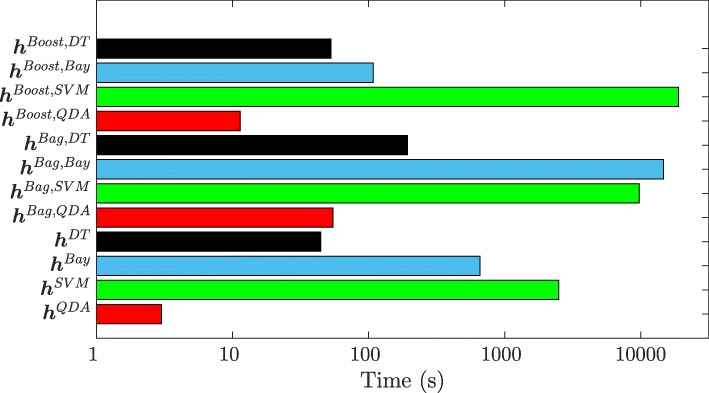
Runtimes for different classifier strategies, averaged over all 25 3FCV runs on training cohort *D*_1_. Colors correspond to different classifier families: QDA (red), SVM (green), naïve Bayes (blue), DTs (black). Note X-axis is log-scaled for display purposes

Figure 5 depicts the computation times for training and evaluating the different classifiers considered in this study for all 3 folds of a single 3FCV run (averaged over all 25 3FCV runs). All computations were performed on 32 or 72 GB RAM quad core 64-bit Intel cluster computers. Note that the X-axis of Fig. 5 has been log-scaled for ease of display.

**h**^*QDA*^ required the least amount of computation time, followed by **h**^*DT*^. **h**^*SVM*^ required the most time for training and evaluating the classifier amongst all algorithms; training and testing times were even longer for **h**^*Bag,SVM*^ and **h**^*Boost,SVM*^. Thia is likely because of the additional grid search required to estimate the SVM parameters *Ω* and *λ*, which significantly increased the amount of time required. Note that SVM classifiers also required more careful memory management for voxel-wise classification, compared to the other methods.

Bagging was seen to typically increase computation time by a factor of 5, while boosting increased the computation time by a factor of 20. This may be because with bagging, the component classifiers are trained on smaller bootstrapped sample subsets, whereas with boosting, the component classifiers are trained on the entire set of training samples.

## Discussion

Our primary findings from this work were the following,

- We identified the most consistently performing method across all 3 sites as the boosted QDA classifier. It had a relatively high AUC (= 0.735) in the training cohort as well as in both validation cohorts (average AUCs of 0.683 and 0.768, respectively). Coupled with its relatively quick execution time (2nd lowest among all methods), we believe this would make it the best classifier overall. Our second choice would be the single QDA classifier, which did not perform significantly worse (average AUCs of 0.730, 0.686, 0.713 for each of the sites) than the boosted QDA classifier.
- The performance of all variants of the decision tree classifier (single, bagged, boosted) were overestimated by ≈10*%*, when compared between the training and validation cohorts. In fact, the top-performing classifier identified in the training cohort was the boosted decision tree classifier (AUC =0.744), but this classifier performed more variably when evaluated on multi-site data. This clearly indicates the need for independent validation when building CAD models, as otherwise these perhaps less generalizable models would have been identified as the top performer.
- The popular SVM classifier achieved reasonable classification performance in the training cohort alone (similar to previous SVM-based PCa detection schemes for prostate T2w MRI [22]). However, they took the longest to train and test, and did not achieve convergence in multi-site validation. This may be a consideration to take into account as prostate CAD schemes start to undergo larger scale multi-site validation.
- We could not reach a clear conclusion regarding which of boosting and bagging yielded better performance across the classifier strategies. There were no significant differences in their performance in multi-site validation.
- Satisfying the conditions of bias and variance were extremely crucial when constructing classifier ensembles. While SVMs and DTs show significant improvements within both bagging and boosting frameworks, Bayesian and QDA classifiers provided a more mixed performance as they suffered from low variance and/or high bias. However, not all of these trends generalized in multi-site validation.
- For all the classifiers considered, performance in the 2 validation cohorts *D*_2_ and *D*_3_ fell within the confidence bounds of their performance in the discovery cohort *D*_1_. Thus, despite heterogeneous acquisition and imaging characteristics across the 3 sites, our post-processing steps (correcting for bias field and non-standardness) appear to have enabled some degree of harmonization in terms of radiomic features and associated classifier models. Appropriate post-processing of multi-site imaging data may therefore be critical when evaluating radiomic classifiers in this fashion.
- In terms of the site-specific performance trends, it is interesting to note that all classifiers performed worse in *D*_2_ than in *D*_3_. While all 3 sites used a 3 T magnet, *D*_2_ had a lower voxel resolution than *D*_1_ and *D*_3_ (which were similar to each other). This seems to indicate that voxel resolution may have a marked effect on classifier performance. This result has also been observed in previous phantom studies of texture analysis in medical imaging [46].

In the context of the specific problem of voxel-wise PZ PCa detection via T2w MRI, we achieved classification accuracies comparable to the literature: optimal AUCs between 0.683-0.768 across 3 different sites. To our knowledge, Rampun et al. [43] have performed the only other such classifier comparison study, where they evaluated 11 different classifiers for voxel-wise prostate cancer detection in the PZ while using 45 patient studies from a single site. While they reported a Bayesian Network classifier as their top performer (AUC = 90.0 ± 7.6), in our experiments both single and ensemble Bayesian classifiers performed well in either the discovery or the testing cohorts (but not both). Dinh et al. [47] utilized 106 patients imaged on scanners from 2 different manufacturers to identify a robust set of statistics from multi-parametric MRI for prostate cancer diagnosis. As opposed to the voxel-wise detection problem examined by us, they developed a linear mixed model to identify which expert-delineated lesions within the PZ had a Gleason score of at least 7 (i.e. a region-wise classification) which achieved per-site AUCs of 0.85 and 0.90. In a more limited study of 18 patients imaged on 2 different scanners [48], a cross-scanner MR intensity normalization technique was presented for detecting which pixels within expert-annotated regions in the PZ corresponded to cancerous or benign. In the current study, we corrected for cross-site intensity drift via histogram standardization [29]; likely due to which all the classifiers performed relatively consistently across the 3 sites. Most recently, a multi-institutional study of radiomic features across 3 different sites (80 patients) was able to identify a PZ tumor-specific set of features for voxel-wise detection of prostate cancer regions [32]. While multi-parametric MRI data was utilized, radiomic features from T2w MRI were most often identified as discriminatory; and they reported comparable AUCs to our own (between 0.61-0.71).

We do acknowledge a few limitations of our study. Despite being one of the first multi-site studies examining voxel-wise PCa detection, our cohort size is still somewhat limited (85 studies across 3 sites). However, this is among the larger cohort sizes when compared to a majority of PCa detection studies in the literature [22] (median cohort size ≈30 studies). The central gland, PZ, and tumor regions were manually annotated on MRI by expert radiologists. While these annotations were done based on corresponding excised pathology images and reports, there may be some error in terms of how precise the delineations are. Notably, such expert annotations have been popularly used in the PCa detection literature [22], perhaps in acknowledgement of how difficult it is to get precise “ground truth” for this problem. However, for two of our cohorts, the expert annotations were only used for independent evaluation of the classifier, potentially lessening the impact of annotation error. With the availability of more comprehensive “ground truth” pathologic information, an avenue of future work could be to identify which radiomic classifiers best generalize for characterizing Gleason grade or benign confounders (e.g. prostatitis) on multi-site MRI data. We also limited ourselves to the use of T2w MRI as opposed to a multi-parametric (MP) MRI exam. The reason for the choice of T2w MRI alone was dictated by non-availability of all MP-MRI protocols across 3 different sites. Additionally, we opted to utilize a specific set of radiomic descriptors of T2w MRI [33] for the construction of PZ-specific PCa classifiers. Additional features may also be employed in this regard, and could be an avenue for future work. We also limited ourselves to empirically comparing 12 classifier strategies. However, based on the 4 distinct types of classifiers considered in this study, we believe our results may be generalized to other classifier families (e.g. relevance vector machines are similar to SVMs).

## Conclusions

In this work, we empirically compared and evaluated 12 different radiomic classifier ensembles derived from 4 classifier families (QDA, Bayesian learners, Decision Trees, and Support Vector Machines), in terms of accuracy and computation time, for voxel-wise detection of prostate cancer in 85 high resolution T2w MRI patient datasets curated from across 3 different sites. A secondary motivation of this study was to investigate whether classifier trends on data curated from a single site generalize to data acquired from different sites and scanners. Our results suggest that simpler classifiers (such as QDA and its ensemble variants) may be more robust, accurate, and efficient for prostate cancer CAD problems, especially in the context of multi-site validation. A more detailed understanding of radiomic feature and classifier trends in large multi-site settings may be crucial for clinical usage of radiomics-based PCa detection on MRI.

## Supporting information: Appendix A

**List of selected radiomic features derived from T2w MRI-** Below, we enumerate the result of feature selection, using data from the training cohort *D*_1_. Parameters incude: (a) for 1st and 2nd order statistics, window size (*WS*∈{3,5,7}); (b) for Gabor wavelets, orientation $\left (\theta \in \left \{0,\frac {\pi }{8},\frac {2\pi }{8},\frac {3\pi }{8},\frac {4\pi }{8},\frac {5\pi }{8},\frac {6\pi }{8},\frac {7\pi }{8},\pi \right \}\right)$ and wavelength (*λ*∈{2.83,5.66,8.20,11.31,22.63,45.25}). Haar wavelets did not have an associated parameter, and were also not selected in the top 25 radiomic features.

1. 2nd Order Statistic Difference Entropy (*WS*=7)
2. Gabor *θ*=1.57,*λ*=45.25
3. 1st Order Statistic Graylevel Median (*WS*=3)
4. Gabor *θ*=0,*λ*=22.63
5. Gabor *θ*=2.75,*λ*=11.31
6. Gabor *θ*=1.18,*λ*=45.25
7. 2nd Order Statistical Energy (WS=7)
8. Gabor *θ*=2.75,*λ*=22.63
9. Gabor *θ*=2.36,*λ*=2.83
10. Gabor *θ*=0,*λ*=2.83
11. 2nd Order Statistic Sum Average (*WS*=3)
12. Gabor *θ*=0.3927,*λ*=11.31
13. Gabor *θ*=1.96,*λ*=45.25
14. 2nd Order Statistic Inverse Difference Moment (WS=7)
15. Gabor *θ*=2.36,*λ*=5.66
16. 1st Order Statistic Mean (*WS*=3)
17. 1st Order Statistic Range (*WS*=3)
18. Gabor *θ*=1.18,*λ*=22.63
19. 2nd Order Statistic Sum Entropy (*WS*=7)
20. Gabor *θ*=2.75,*λ*=5.66
21. Gabor *θ*=1.96,*λ*=8.20
22. Gabor *θ*=0,*λ*=5.66
23. 2nd Order Statistic Difference Entropy (*WS*=3)
24. Gabor *θ*=2.35,*λ*=8.20
25. Gabor *θ*=2.75,*λ*=2.83

## Abbreviations

3FCV: Three fold cross validation
ANOVA: One way analysis of variance
AUC: Area under the ROC curve
CAD: Computer aided detection
DA: Discriminant analysis
DT: Decision tree
IRB: Institutional review board
K-W: Kruskal-wallis
MAD: Mean absolute deviation
MP: Multi-parametric
MRI: Magnetic resonance imaging
MSV: Multi-site validation
PCa: Prostate cancer
PZ: Peripheral zone
ROC: Receiver-operating characteristic (curve)
SVM: Support vector machine
T2w: T2-weighted
